# Last twenty-years activity of cardiovascular tissue banking in Barcelona

**DOI:** 10.1007/s10561-022-10059-9

**Published:** 2023-02-27

**Authors:** C. Castells-Sala, M. L. Pérez, E. Agustí, A. Aiti, E. Tarragona, A. Navarro, J. Tabera, O. Fariñas, J. L. Pomar, A. Vilarrodona

**Affiliations:** 1 Barcelona Tissue Bank, Banc de Sang i Teixits (BST), Barcelona, Spain; 2 Organització Catalana de Trasplantaments (OCATT), Barcelona, Spain; 3 Institute for Cardiovascular Diseases. Hospital Clinic de Barcelona, University of Barcelona, Barcelona, Spain; 4 Biomedical Research Institute (IIB-Sant Pau; SGR1113), Barcelona, Spain; 5 Vall Hebron Institute of Research (VHIR), Barcelona, Spain; 6 Barcelona Tissue Bank (BTB), Banc de Sang i Teixits (BST, GenCAT) Passeig Taulat 116, E-08005 Barcelona, Spain

**Keywords:** Cardiovascular, Homograft, Heart valves, Vascular segments, Banking, Tissue establishment

## Abstract

The Barcelona Tissue Bank was established from the merge of two previous multi-tissue banks. Potential donors are screened by Donor Center staff and multi-tissue retrieval is performed by specialized own teams. Tissue processing and preservation is performed in clean room facilities by specialised personnel. After quality control of both donor and all tissues results, the heart valves and vascular segments are stored until medical request. The aim of this report is to present the cardiovascular tissue activity and retrospectively evaluate the outcomes of the changes performed in last 20 years. Cardiovascular tissue from 4088 donors was received, specifically 3115 hearts and 2095 vascular segments were processed and evaluated. A total of 48% of the aortic valves, 68% of the pulmonary valves and 75% of the vascular segments were suitable for transplant. The main reason for discarding tissue was macroscopic morphology followed by microbiological results, for both valves and arteries. Altogether, 4360 tissues were distributed for transplantation: 2032 (47%) vascular segments, 1545 (35%) pulmonary valves and 781 (18%) aortic valves. The most common indication for aortic valve surgery was the treatment of endocarditis, while for pulmonary valves, it was congenital malformation reconstruction. Vascular segments were mainly used for reconstruction after ischemia. During this period, a number of changes were made with the goal of enhancing tissue quality, safety and efficacy. These improvements were achieved through the use of a new antibiotic cocktail, increasing of donor age criteria and changing the microbiological control strategy.

## Introduction

The advent of cryopreservation, and the experience reported by O’Brien and his group from Brisbane (Australia) in 1987, represented a critical step forward in the creation of cardiovascular (CV) tissue banks worldwide. The precise value contribuded by the studies performed by O’Briens’s group was to show that all stages of the process, including procurement, manipulation and preservation, are vitally important for the long-term performance of transplanted tissues (O’Brien et al. 1987).

The advantage of being able to keep the tissues cryopreserved, made it possible to schedule surgeries and store a range of homografts of different sizes, as well as increase the safety of those tissues. Moreover, the increasing interest shown by CV surgeons led to the need for the adequate organisation of tissue donation and banking, in an attempt to cope with the demands of both patients and surgeons. Today, there are 99 tissue establishments (TE) in the European Union Member States authorised for the procurement, processing and/or preservation of CV tissues (European Comission). Different TEs were organized by the State Members during the 1980’s (Goffin et al. 1996; Jashari et al. 2010). Specifically, in 1989 the Cardiovascular Bank (Criobarna Project) started operating at Hospital Clínic de Barcelona (HCB) (Mestres et al. 2000) thanks to the collaboration between the Departments of Cardiovascular Surgery, Microbiology, the Hospital Pharmacy, the Transplant Coordination Unit and several required grants including a 50% of the necessary capital from the Spanish Ministry of Industry (CEDETI) and the remaining from funds of many local institutions like the City Hall, the University of Barcelona, the HCB and investments from the related industry for liquid nitrogen facility.

The first aortic valve (AV) replacement with a fresh homograft preserved in antibiotic solution performed in HCB was carried out in January 1989 in a patient with active infective endocarditis (Martínez et al. 1991; Mestres; et al. 1991). Cryopreservation was introduced in late 1990 and after implementation of an effective decontamination cocktail, cryopreservation and thawing processes, transplantation of cryopreserved homografts started succesfuly. A patient underwent tricuspid valve replacement in 1991 with a cryopreserved mitral homograft because of infective endocarditis and HIV infection (Mestres et al. 2006; Pomar et al. 1994). In October 1992, the first transplantation of a cryopreserved artery was performed in a patient with a mycotic aorta-cava fistula.

**Fig. 1:**
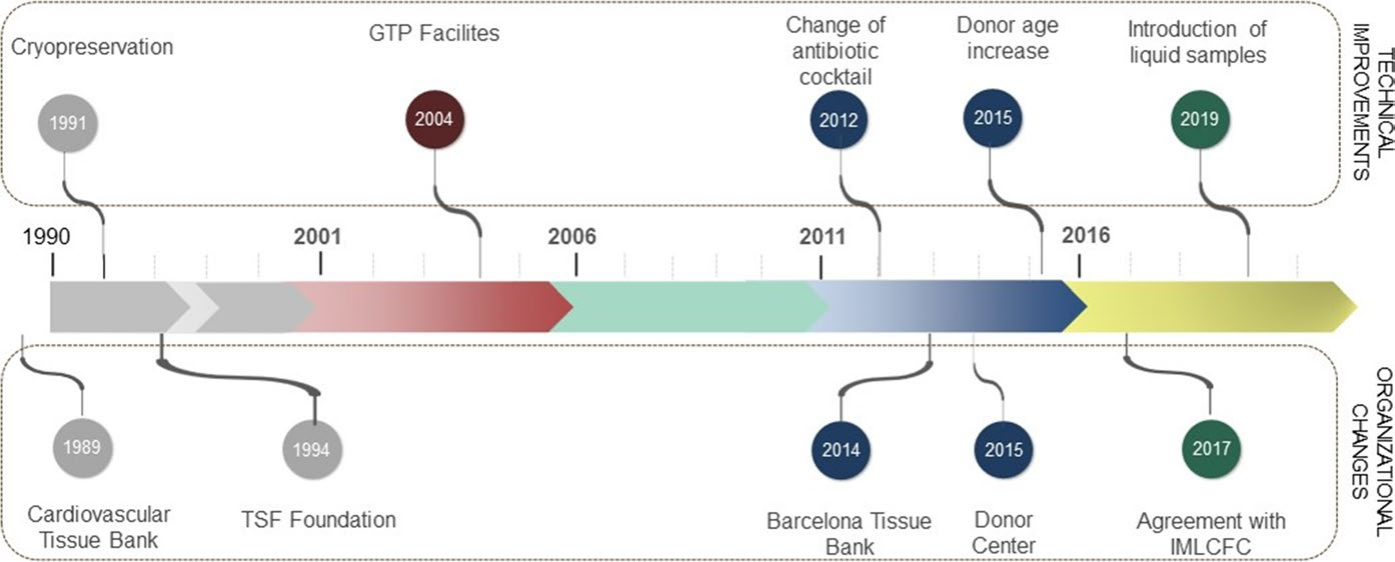
Time line of CV tissue bank in Barcelona

Between 1989 and 1994, the CV tissue bank was fully integrated in HCB. In June 1994, the Transplant Services Foundation (TSF) was created by the HCB to serve as a non-profit organisation to coordinate all organ and tissue donation and transplantation activities of the hospital. The merger of the hospital’s eye bank, bone bank, CV bank and skin bank under the TSF umbrella allowed to establish joint protocols for donation, retrieval, processing, storage and release for transplantation, optimizing resources with the aim to improve clinical options for the patients. In the following years, TSF improved tissue bank methodologies and facilities culminating in 2004 with the construction of a clean room facility specifically designed to work under GMP (Good Manufacturing Practices) and GTP (Good Tissue Practices) conditions in a new location (Fig. 1).

In parallel, since 2008 certain tissue banking activity was carried out by the blood and tissue bank (BST, Banc de Sang i Teixits) centralized at that time in Vall d’Hebron Hospital. In 2014, the Catalan Ministry of Health ordered merging the two tissue banks sharing the same activity, resulting in the current Barcelona Tissue Bank (BTB). In addition, today, management of all tissue donors from Catalonia has been unified thanks to the creation of the Donor Center (DC) in 2015, which is responsible for screening all potential donors and for searching for new sources of donors, such as from the Institut de Medicina Legal i Ciències Forenses de Catalunya (IMLCFC), with which an agreement was signed in 2015 (Fig. 1). In 2017 the activity of CV tissue retrieval started in IMLCFC.

During these years, different technical improvements have been implemented at the CV tissue bank, starting with tissue cryopreservation and processing in clean rooms (Fig. 1). A few years later, in 2012, the decontamination protocol used from early 1990s was replaced by a new antibiotic cocktail and, three years later, the donor age limit for heart donors was increased from 65 to 70 years old, in an attempt to increase the number of preserved tissues. In 2019, the microbiological sampling strategy was improved by introducing the analysis of liquid samples, with the aim of detecting any possible contamination (Fig. 1).

This paper aims to review the activity of the last 20 years, from 2001 to 2021, in banking of heart valves and vascular segments for transplantation in Barcelona, with particular focus on the changes made in the bank seeking a continuous quality improvement. The data referring to donation, processing and distribution have been analysed.

## Materials and methods

### Organisation and structure

BST is a public agency under the Catalan Ministery of Health. Its mission is to guarantee the supply and proper use of human blood and tissues in Catalonia, following applicable regulation (Directive 2004/23/EC 2004; Commission directive 2006/17/EC; Commission Directive 2006/86/EC) and good practices (EDQM; Council of Europe 2019).

The BTB is subjected to Spanish Legislation (RD-L 9/2014 2014) and European Directives on the use of tissues and cells of human origin for therapeutic purposes (EU 2004/23/EC). The BTB’s quality standards are in accordance with European Directives 2006/17/EC and 2006/86/EC, as well as the *Guide to the Quality and Safety of Tissues and Cells for Human application* (EDQM, 4th Ed.).

The BTB is a multi-tissue bank with two main working areas. On one hand, there is a DC, which is responsible of (1) complete donor screening (attending potential donor calls from any hospital in Catalonia or Spain, consent interviews with the families, judicial consent if applies and donor testing), (2) management of the retrieval teams, and (3) tissue procurement. On the other hand, there is a tissue establishment (TE), which is responsible for preparing, evaluating, storing and distributing tissues for transplantation.

DC is composed by medical and nursing staff and also includes a multi-tissue retrieval team, which is always composed by a doctor (team leader) and two technician that can be nurses or professionals with other qualifications in the health field. In the TE, the people responsible for the preparation and cryopreservation of CV tissue are technicians specialised in tissue dissection and evaluation (CV but also skin, amniotic membrane, ocular and muskulosqueletal tissue); in addition, there is also a research team. The BTB team also includes quality assurance and quality control staff. Finally, in the TE there is a tissue allocation team in charge of receiving all the requests from transplant centres as well as tissue shipment arrangements. To guaranty process traceability it is required an implant confirmation form and the information of the final use of the tissue.

### Donation and retrieval

Heart beating (brain death), non-heart-beating and exitus donors are screened for heart valves and vascular segments donation. A maximum warm ischemia time of 24 h is accepted if the body has been refrigerated within 6 h after asystolia, or 12 h if the body has not been refrigerated. Hearts from living donors (who have received a heart transplant) can also be obtained.

Complete donor evaluation is performed in order to discard any general or specific contraindication for CV donation. This evaluation includes, but is not limited to, a review of the medical and social history, serological and microbiological testing for transmissible diseases, physical examination of the body, autopsy findings if apply, as well as any other relevant information provided by relatives or team leader responsible for the retrieval. Compulsory serology testing includes HIV, hepatitis B and C, syphilis, HTLV and nucleic acid determination of HIV, hepatitis B and C, and nowadays also hepatitis E. Additionally, depending on the country of origin of the donor, the information obtained from the relatives, the travel history and the epidemiologic situation, this serology could be completed with additional determinations such as Trypanosoma Cruzy or West Nile Virus.

After complete donor evaluation and next of kin donation consent (or donor himself if living donation), tissue donation is performed. Tissue retrieval is always performed in an operating room (OR). BTB has the possibility of performing the retrieval in diferent locations: (a) specific facilities for tissue recovery located in two diferent hospitals and IMLCFC; (b) OR in hospitals authorized for tissue donation; or (C) transplant hospitals in case of living donation.

CV tissue is procured by the multi-tissue retrieval teams but, in heart-beating organ donors, the tissue can also be procured by the organ transplant team.

In multi-tissue donors, CV tissues are recovered simultaneously to muskulosqueletal, opening the thoracic cavity by esternotomy and the abdominal cavity, after ocular and skin tissue retrieval. Heart is retrieved preserving the aortic arch and pulmonary branches as long as possible. Standard recovered vascular segments are aorto-iliac bifurcation and femoral arteries, but other segments from descending thoracic aorta to abdominal aorta can be also recovered depending on the needs.

After recovery, CV tissues (heart and vascular segments) are packaged separately into sterile containers with isotonic solution (ringer lactate, saline solution), each one wrapped in sterile bag and kept in a thermobox to ensure a temperature between + 2/+8ºC. The container is labelled with Single European Code (given by the IT system in the DC) and forwarded to TE, where it is processed not later than 32 h after retrieval. The tissue is accompanied by documentation that guarantee full traceability, including the retrieval form with some compulsory identification data: donor ID, donation time and date, asystolia and retrieval data.

### Tissue processing

The first step upon arrival is verification of the package, labelling and transport conditions of the container and the retrieval form. CV tissue processing, evaluation and preservation is performed in a specialised clean room facility. All steps of the protocol are performed under laminar flow Class A, in Class B environment. After dissection of the heart, the tissues are rinsed and macroscopically evaluated looking for atheroma, calcification, aneurism, fenestrations and petechial presence which may cause the tissue discard. Also any significant finding is recorded. Valvular ring and distal artery diameters are measured using calibrated Hegar dilators as well as the length of the aortic and pulmonary arteries. Moreover, a coaptation test is performed filling the conduit with media and looking for leakage to ensure proper performance of the valve. As part of the preparation of the arteries, fat is removed, an inspection for atheroma, calcification or iatrogenic damage is performed and total length and proximal and distal diameters are measured. A photograph of each tissue is taken.

When there is no discarding feature in the tissue, the validated antibiotic cocktail is prepared containing amikacyn (Normon Laboratories - Spain; 791,301), metronidazole (B. Braun Medical SA- Spain; 600,496), ciprofloxacin (Altan Farmaceuticals, S.A.; 643,494), vancomycin (Lab. Reig Jofre, S.A; 606,390) and amphotericin (Xalabarderfarma - Spain) in RPMI (Roswell Park Memorial Institute Medium, Corning, 15,040 CV). The grafts are immersed for overnight decontamination at room temperature. After decontamination, a macroscopic quality control is performed and the grafts are prepared for cryopreservation in a double cryo-bag with cryoprotectants, namely dimethylsulfoxide (DMSO, WAK CHEMIE, WAK DMSO 10) and human albumin (Grifols, 726,609,670,612) in RPMI 1640. Finally, controlled freezing is carried out into a biological chamber (Carburos Matálicos CM2010) in order to decrease the temperature to − 110 °C. Long term storage of the cryopreserved homografts is performed in liquid nitrogen tanks at − 196 °C for 5 years.

Microbiological samples are taken during preparation of the tissue and packaging steps. Biopsy samples taken from the tissue before packaging are included in thioglycollate tubs (Becton Dickinson, 221,787) for the study of bacteries and fungi. Liquid samples from transport media and preservation media are inoculated in BD BACTEC™ PLUS-Aerobic/F Medium (BD Bioscience, 442,192) and BD BACTEC™-Lytic/10 Anaerobic/F Medium (BD Bioscience, 442,265) for aerobic and anaerobic growth and fungi detection. Sistematically, after dissection all hearts are evaluated macroscopically and histologically by the Anatomical Pathology Service of HCB.

### Quality assurance and quality control

Homografts stay in quarantine until all test results from donor and processing are reviewed. Quality controls for the tissue suitability include: environmental controls of the facility, microbiological testing, macroscopic evaluation of the tissue as well as pathologic anatomy report from the rest of the heart.

Final tissue release is given by the medical doctor in charge of quality control (Responsible Person) after checking all donor and tissues results. For each tissue distributed, TE provides the next documentation: donor and tissue report, handling and thawing instructions, implant confirmation, follow-up and vigilance notification forms.

### Data source

Data regarding donation, retrieval, processing and distribution were obtained from TSF reports (donors 2001–2007), TSF and Vall d'Hebron Hospital records (donors 2008–2014), and BTB reports (donors 2015–2021).

### Statistics

The PRISM software version 5.00 (GraphPad Software, San Diego CA, USA, www.graphpad.com) was used for statistical analysis. All results are presented as the mean ± standard deviation (MD ± SD). The non-parametric two-tailed Mann-Whitney test was used and p values less than 0.05 were considered statistically significant.

## Results

### 1. Donation

**Fig. 2:**
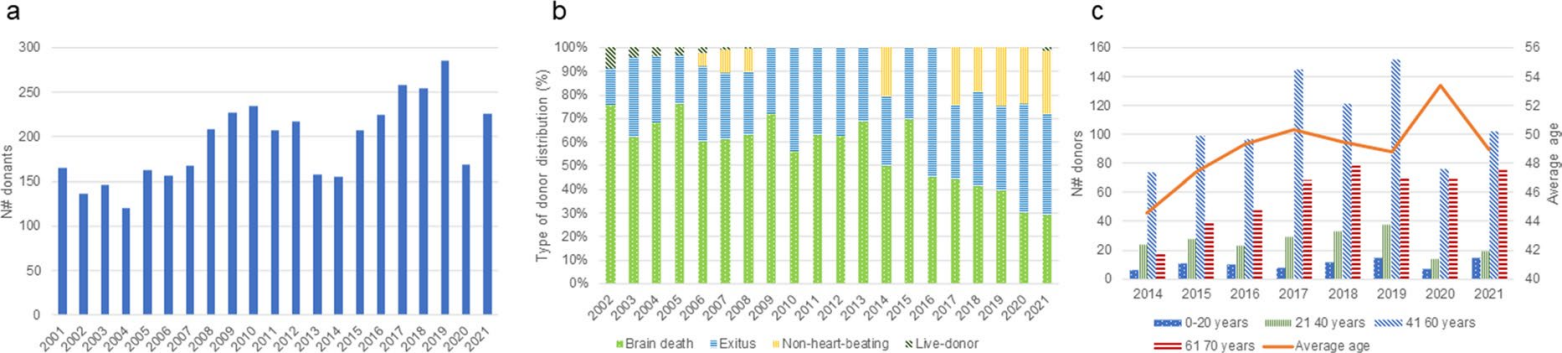
Tissue donation. **a** Number of donors received per year; **b** Percentage of type of donor per year; **c** Donor age distribution

During the last 20 years of activity, the CV tissue bank received a total of 4088 donors. From these donors, 3115 hearts and 2095 vascular segments were obtained (distribution per year presented in Figs. 2a and 4a, and 4b). As can be observed in Fig. 2a, the number of donors per year fluctuated between 120 and 2004 and 286 in 2019, with two donation peaks in 2010 and 2019 and three valleys in 2004, 2013–2014 and 2020.

As presented in Fig. 2b, in the early years of the CV bank, tissues were obtained mostly from brain-death donors, representing 64.93 ± 7.39% of the donors during the first decade, but decreased significantly to 38.33 ± 7.06% during the second decade (*p* < 0.05). On the other hand, exitus donors represented 30.14 ± 7.02% of the CV donors during the first decade, while during the second decade they increased significantly up to 42.00 ± 8.07% (*p* < 0.05). Interestingly, while during the first decade there was a significant difference between the CV tissues obtained from brain death and exitus donors (*p* < 0.0001), nowadays this difference is not statistically significant. The percentage of non-heart beating donors also increased significantly after 2011 (*p* < 0.005).

Demographically, between 2014 and 2021, most part of the donors aged between 40 and 60 years old, accounting for 53% of the total donors. As it can be observed in Fig. 2c, the number of older donors tends to increase during the last 8 years. The average age between 2014 and 2021 was 49.0 ± 2.5 years, without any significant differences. The donors’ distribution did not change during the last 20 years, being 67 ± 4% male donors and 33 ± 4% female donors (*p* < 0.0005).

The received donors came mainly from Catalan hospitals (90.10%), but there are also some donors from the rest of Spain (8.99%) (data not shown).

**Fig. 3:**
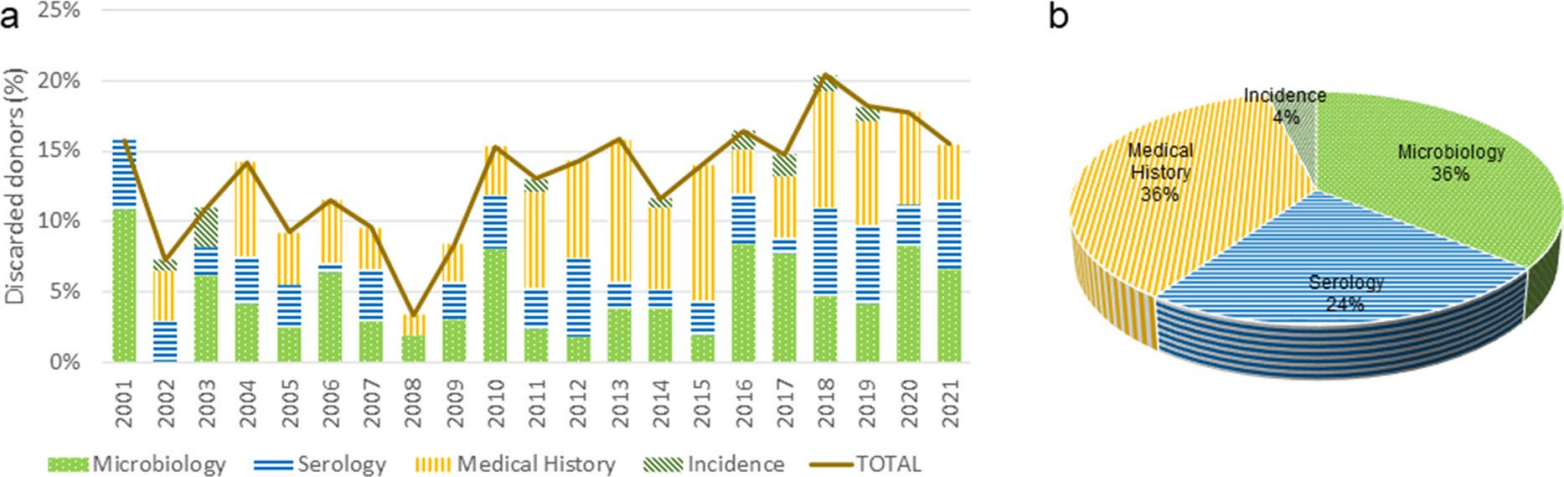
Discarded donors. **a** Percentage of discarded donors per year and the reasons of discard; **b** Causes of discaring during period 2001–2021 in BTB.

Four main reasons for donor discard can be identified: serological results, medical history including the evaluation of any finding during retrieval, microbiological testing before decontamination and any technical incidence with the tissue before arrival at the TE (e.g., packaging and transport temperature). During these 20 years of activity, the rate average of discard related to the donor has been 12.85%. The lowest rate of donor discard was achieved in 2008 with a 3.35%, while the highest was achieved in 2018 with a 20.39%. After the peak in 2018, the donor discard rate has remained stable between 15 and 20%, with a tendency to decrease (see Fig. 3).

Regarding donor discard causes, 37.05 ± 17.53% was due to medical history, 36.06 ± 18.12% for positive microbiological donor testing, 23.16 ± 11.40% because of serological results, and 3.72 ± 6.18% for technical incidence, such as temperature maintenance. No statistically significant differences were observed during all analysed period.

#### 2. Tissue processing

**Fig. 4:**
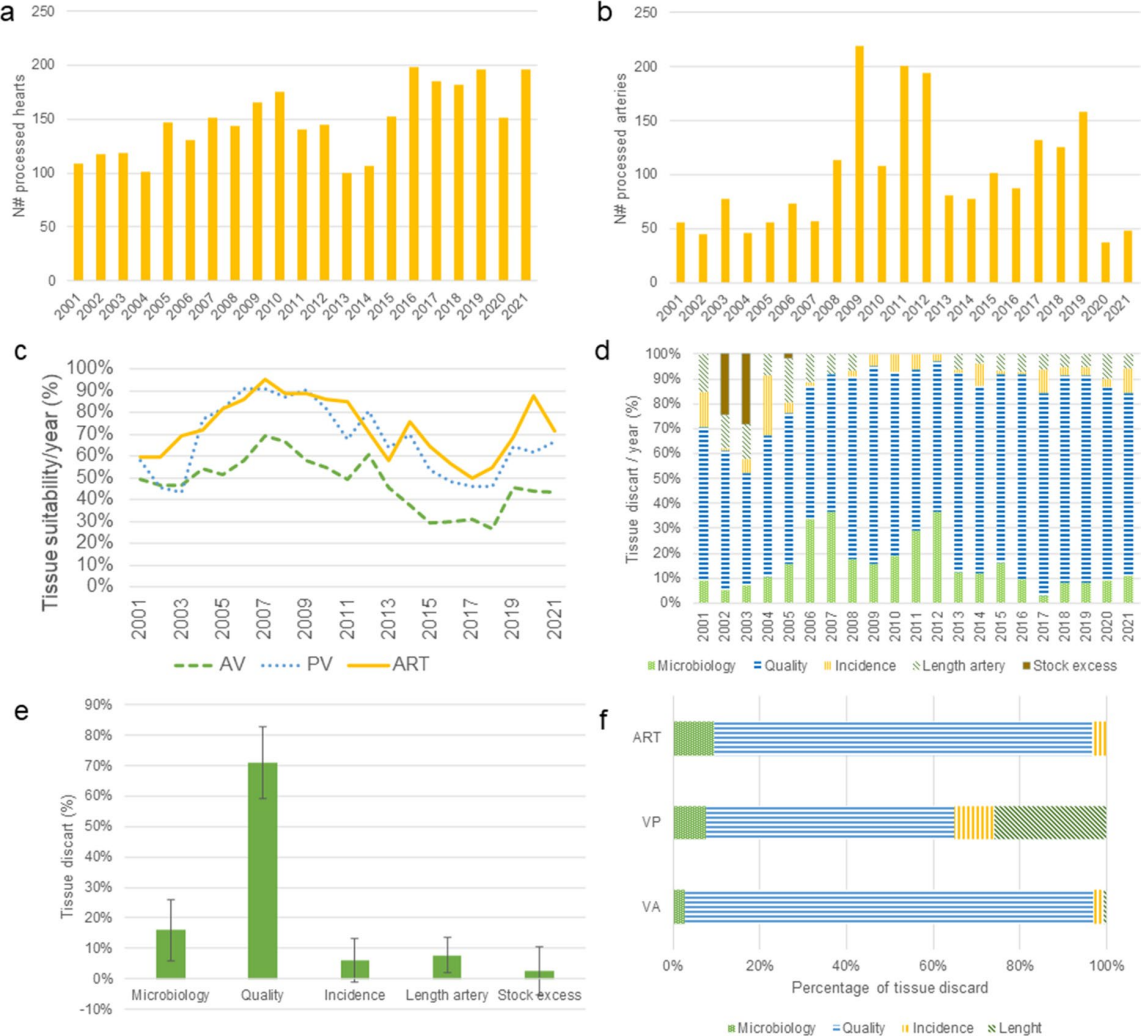
Tissues processed and their suitability for implant. **a** Number of heart valve donors processed per year; **b** Number of vascular segments donors processed per year. **c** Reason for tissue discard per tissue type and year; **d** Causes of tissue discard per year normalised by total of non-viable tissues during the year; **e** Causes of tissue discard; **f** Percentage of tissue discard causes per tissue type during the period of 2014–2021

During the period 2001–2021, all retrieved hearts were processed under laminar flow (in clean rooms from 2014) and carefully evaluated to guarantee tissue suitability for transplantation purposes. From the 3115 hearts received, 48.52 ± 10.48% of the AV and 68.85 ± 15.07% of the PV were suitable for transplant, while from the 2095 vascular segments donors, 75.78 ± 12.11% of the tissues were suitable for transplant. The percentage of tissue suitability was studied. As shown in Fig. 4c, the percentage of AVs accepted for transplantation ranged between a minimum of 29.41% in 2015 and a maximum of 69.13% in 2007; the percentage of PV accepted ranged between a minimum of 43.52% in 2003 and a maximum of 91.03% in 2007; and the percentage of vascular segments accepted ranged between 56.36% and 2016 and 95.18% in 2007.

As it can be observed in Fig. 4d, the main reason for CV tissue discard, both for valves and vascular segments, was the morphology in 71.09 ± 11.77% of the cases (atheroma and/or calcifications). The second reason for tissue discard was positive results in the microbiological controls during the processing and the cryopreservation (15.91 ± 9.91% of discarded tissues). The remaining discarded tissues were not suitable for transplantion due to short artery length (7.62 ± 5.81%), processing errors (6.10 ± 7.21%) and stock excess (2.59 ± 7.90%) exclusively in the years 2002 and 2003.

Taking into account the tissue type, most of the AV were discarded due to morphology (94.22%), while the rest were discarded due to positive microbiological result (2.57%), incidents during processing (2.41%), or artery lenght (0.80%). On the other hand, tissue quality accounts for 57.19% of the tissues discarded in the case of PV. The second reason for PV discarding was artery length, accounting for 26.03%, followed by processing errors (9.25%) and positive microbiology results (7.53%). In the case of arteries, 87.08% of the tissues were discarded due to quality, followed by positive microbiology results (9.55%) and incidents during processing (3.37%).

**Fig. 5:**
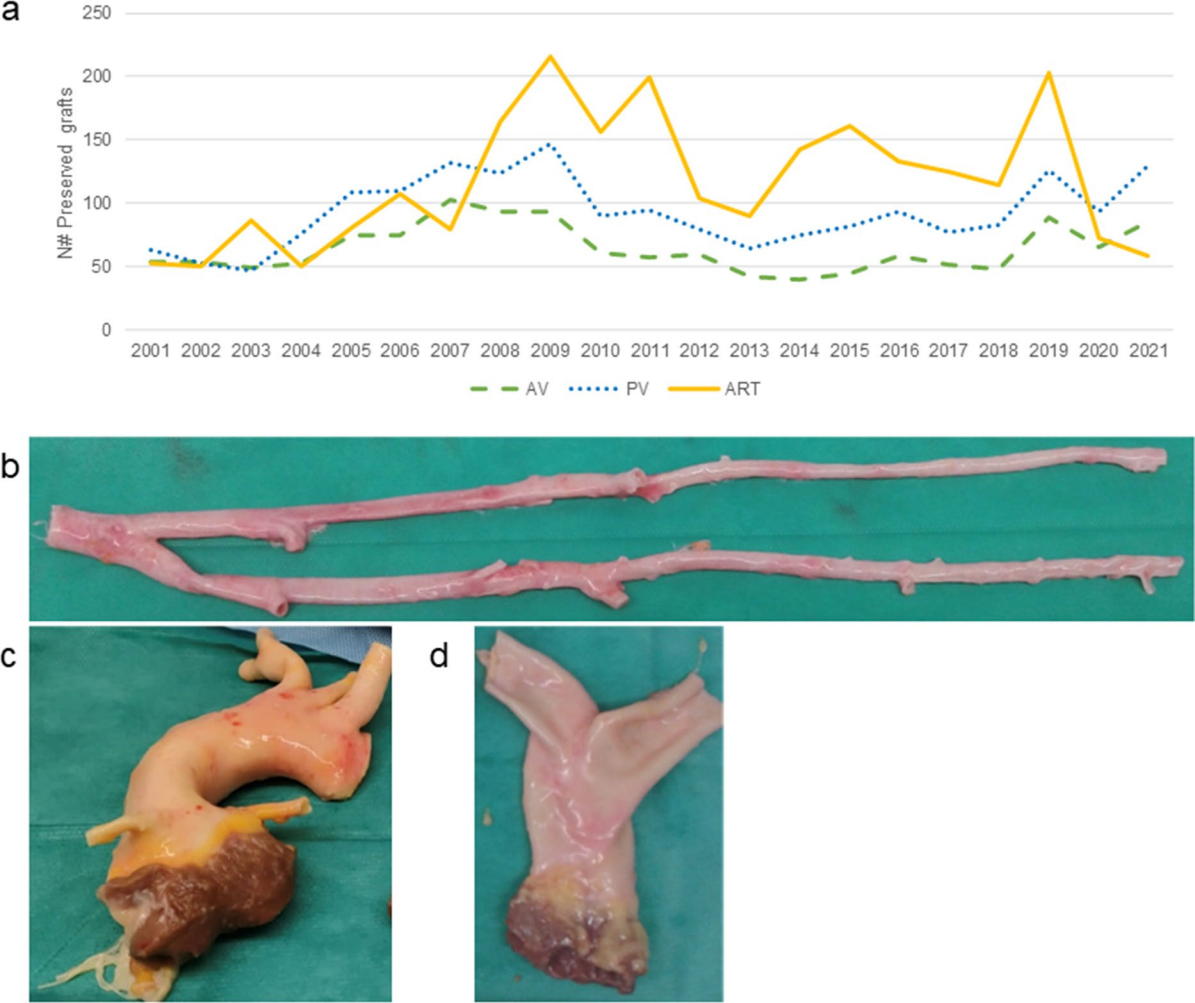
Tissue preservation, **a** Tissue preservation per tissue type and year; **b** Tissues preserved during the period 2001–2021 at the BTB; **c** Aortoiliac bifurcation; **d** Aortic Valve; **e** Pulmonary valve

During these years a total of 1950 PV (34% of preserved tissues), 1353 AV (23% of preserved tissues) and 2446 vascular segments (43% of preserved tissues) were accepted for transplantation (Fig. 5).

#### 3. Tissue distribution

**Fig. 6:**
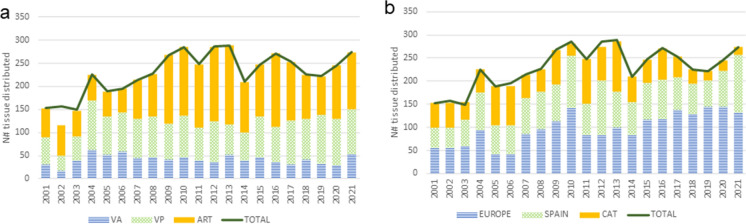
Distribution of CV tissue. **a** Tissue distribution per tissue type and year; **b** Tissue distribution per destination and year

During the last 20 years, a total of 4360 CV tissues have been distributed for implantation (see Fig. 6). Nearly the half of the distributed tissues were vascular segments (47%−2032 grafts), followed by PV (35%−1545 grafts) and AV (18%−781 grafts). The distributed tissues were sent to different hospitals in Catalonia (23%), other regions in Spain (33%) and Europe (44%).

**Fig. 7:**
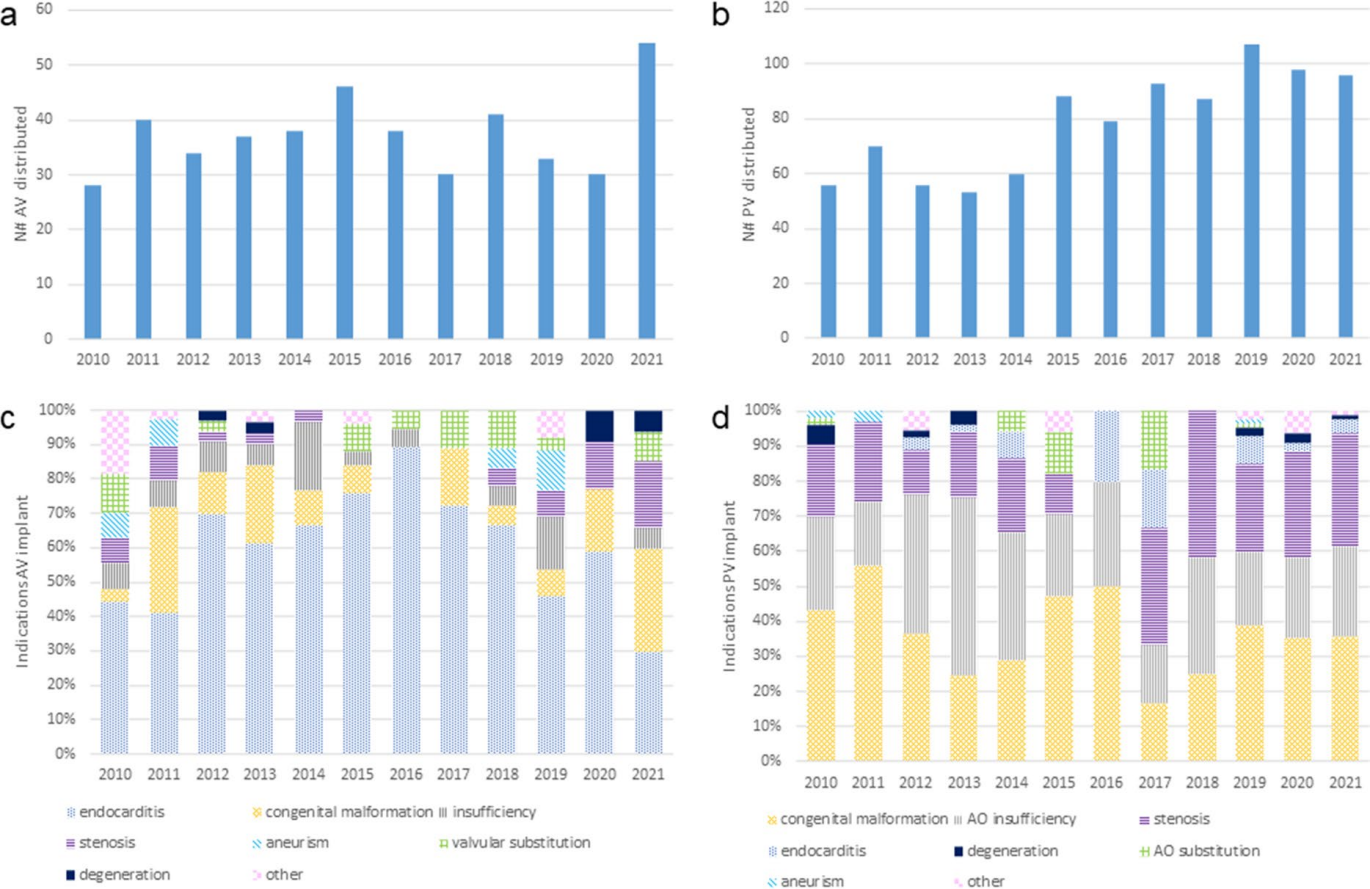
Heart valves distribution from 2010 to 2021. **a** AV distribution; **b** PV distribution; **c** Reported indications for AV implants; **d** Reported indications for PV implants

From January 2010 to December 2021 a total of 1492 heart valves (467 AV and 1025 PV) were distributed for transplantation. AV distribution ranged between 30 and 40 grafts each year until 2021, when more than 50 grafts were requested and provided (Fig. 7a). PV distribution has increased in the recent years from 50 grafts (2010–2014) to 80 (2015–2018) and subsequently to 100 grafts per year (2019–2021) (Fig. 7b).

The main indication for the AV implantation was the treatment of endocarditis with severe complications (56%), followed by congenital malformation reconstruction (16%) and aortic insufficiency (8%) (Fig. 7c). The main indication requiring PV transplant was the reconstruction of congenital heart malformation (38%), followed by AO insufficiency (29%), and stenosis (24%) (Fig. 7d).

**Fig. 8:**
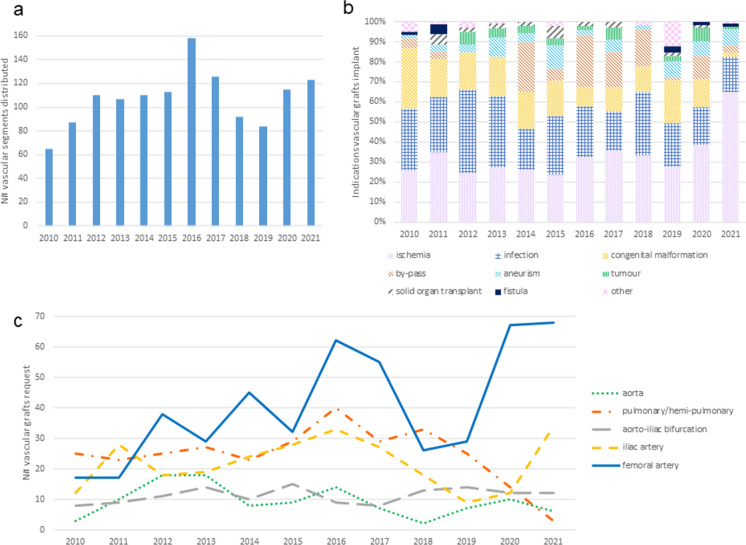
Vascular segments distribution from 2010 to 2021. **a** Vascular segments distribution; **b** Reported indications for vascular segment implants; **c** Evolution of the vascular grafts requests depending on type of graft

During the same period, 2010–2021, a total of 1539 vascular grafts were distributed for transplantation (Fig. 8a): 16% were used for cardiac reconstructive surgery (pulmonary patches for congenital malformations) and 84% for vascular surgery (from which 34% correspond to iliac-femoral grafts for ischemia reconstruction) (Fig. 8b). Interestingly, vascular homografts for reconstruction after ischemia have doubled in 2021 compared with previous years. Together with surgeries triggered by infection, these are now the two main indications for femoral arteries. Distribution of vascular grafts grew from the beginning of TE until 2016, when the demand of pulmonary, femoral and iliac arteries started to decrease. In 2020 and 2021 the distrbution of peripheral arteries recovered to the same levels as 2016, but pulmonary arteries continued to decrease with no recovery (Fig. 8c). During this decade, the percentage of vascular segments distributed is the following: 38% femoral arteries, 23% pulmonary/hemipulmonary patches, 20% iliac arteries, 10% aortoiliac bifurcation, and 9% aortas (ascending or descending aorta).

Since 2008 a Biovigilance (BV) registry was implemented in Catalonia to detect Serious Adverse Events (SAE) and Reactions (SAR) in tissue transplantion. The total number of biovigilance notifications received in the Catalan tissue BV system from 2008 to 2020 was 158 (118 SAE concerning donation and 36 SAR in tissue recipients). From the 36 SAR, 4 (10%) were from CV recipients, 2 heart valves (1 PV and 1 AV) and 2 arteries (iliac-femoral). This data is similar to the European Biovigilance 2019 report where cardiovascular reactions were 15% of the total SAR. The two heart valve reactions detected at the moment of implantation were graded with imputability certain and related to the quality of the homograft, even though any anomalies from the donor or tissue before cryopreservation were detected. Regarding vascular tissue, two patients presented graft failure on day + 5 and + 20 after transplantation and the imputability was classified as probable. Again, donor characteristics and tissue processing data did not reveal any deviation. There was no infection disease transmission through CV tissue transplantation during this period in BTB, whereas it was reported a 11% of infection disease transmission in the European Biovigilance report and the Notify Library (Petrisli et al. 2021). Taking into account the period 2008–2020, the incidence of SAR in CV patients  was 1 SAR for every 975 heart valve transplanted and 1SAR out of 828 artery’s recipient.

## Discussion and conclusion

This review sumarize the activity of the CV bank during the last 20 years (from 2001 to 2021), focusing on the changes implemented to improve tissue quality and patient wellbeing. The activity of CV tissue banks in Europe has increased and the activity of the CV tissue bank of BTB has not been an exception. The general strategy seems to be to centralise the activity in specialised TEs due to the stricter quality and legal requirements for the activity. For instance, the UK banks merged into NHSBTS-TE in 2005, the Netherlands tissue banks merged into ETB-BISLIFE in 2018 and Catalan tissue banks merged into BST in 2014.

The main objective of donation is to respond to the clinical needs, increasing the number of CV tissue donors. According to the EDQM reports of 2003, 1169 hearts for heart valves and 473 blood vessels were retrieved from cadaveric donors in the Member States during that year. This number has increased to 1439 hearts for heart valves and 7347 blood vessels in 2021 (EDQM Newsletter 2004 and 2022). Different TEs reported their activity with variable results: Berlin Homograft Bank (BHB) reported 927 hearts in the period between 2000 and 2009 (Delmo Walter et al. 2012), while the European Homograft Bank (EHB) reported an average of 2021 hearts per decade in the last three decades from the donation network in Belgium, France, Luxemburg, Netherlands, Germany and Switzeland (Jashari 2021); and the Brazilian Human Valve Bank reported a total of 2149 hearts received in the period between 2004 and 2014 (Ferreira et al. 2016). In our case, although the number of donors per year fluctuated between 120 donors and 286 donors, there is a tendency to increase from 1725 retrieved donors in the first decade (x̄ = 172.5 ± 38.5 donors per year) to 2363 donors retrieved during the second decade (x̄ = 214.8 ± 42.1 donors per year). In 2014, two main changes were implemented in BTB (Fig. 1): the merger of the two CV banks in Catalonia and the expansion of age criteria for heart-valve donors from 65 to 70 years (keeping the maximum age for arteries at 55 years old). These two changes impacted directly on the number of heart-valve donors, increasing from 107 heart-valves in 2014 to 198 heart-valves in 2016, which represents an increase of 42.99% in the first year and 29.41% increment in the second year. As the activity of both TSF and BST is included in the data since 2008, the increased number of donors from 2014 could be correlated with the expansion of age criteria. After 7 years applying this criteria, a significant increase has been observed in donor discard from 11.45 ± 3.36% to 16.71 ± 2.22% (*p* < 0.005), mainly due to medical history findings which increased from 1.96 ± 2.42% to 6.03 ± 2.64% (*p* < 0.005). The modification of upper age limit was also internally validated by the EHB in 2016 for PV (Jashari 2021). It is also important to mention the TE logistics. In 2008, the year with the lowest ratio of donor discard, the number of donation centers were low, and the retrieval team was limited and stable. After the merger in 2014, the number of centers and the members of the retrievel team were incremented. This fact implied the need of a training plan with the corresponding learning curve for the selection, evaluation and retrieval. This plan was of outmost importance to perform a proper donor evaluation and relatives interview to detect any possible reason for donor rejection before tissue retrieval, and decrease the percentage of donor discard.

Another important implementation was the creation of the DC in 2015, a single entity in Catalonia, which centralizes all tissue donations. From then on, all exitus donors ocurring in hospitals were reported to the DC, increasing significantly the percentage of exitus tissue donors from 30.14 ± 70.30% to 42.00 ± 8.08% after creation of the DC (*p* < 0.008). This could perhaps explain why the type of donor has changed in the last 20 years from 75% brain death in 2001 to 25% brain death in 2021, as Fig. 2b shows. Regarding non-heart-beating donors a significant increase was observed in the second decade analysed in this study. Non-brain death donors include organ and tissue donors after cardiac arrest (non-heart-beating) and exitus. So, the increase in the number of donors can be attributed to the effort made by the hospitals and national competent authorithy (Organización Nacional de Trasplantes, ONT) to increase the number of organs available for transplantation. For instance, the implementation of the controlled Maastricht III donation programme in Catalonia (Thoung et al. 2016) and the organisational change brought about the creation of the protocol in collaboration with the local competent authority (Organització Catalana de Trasplantaments OCATT).

Demographically, as shown in Fig. 2, most of the donors (67 ± 4%) were male, and this has not changed significantly over the years. During the last 8 years, the majority age group was 40–60 years old (53%), with the average age being 49 ± 2.43 years. Similar sex and age patterns were observed at the Brazilian Human Valve Bank where only 34.5% of the donor were female and 51.5% of the donors were over 36 years old (Ferreira et al. 2016). On the other hand, the Canadian vascular graft bank reported an average donor age of 35 years, with 78% of men donors (Georges et al. 2021).

Unfortunately, during 2020, due to the COVID-19 outbreak, donations ceased for 4 weeks, decreasing by 94.7% compared with the same period in 2019 (Piteira et al. 2021); ten months later donations seemed to have returned to previous levels and have remained stable. Also transplantation activity decrease during this period in Spain (Domínguez-Gil et al. 2020). However, the impact on CV tissue donors was remarkable, reducing donations from 286 to 2019 to 169 in 2020, representing a 23% decrease in heart donations and a 77% reduction in vascular segment donation. This decrease in heart-valves donations is directly related to the pandemic situation, and fortunately, in 2021, the number of heart donors increased again to the level of 2019 (Fig. 3a). The TE and the health services should assess the SOHO supply chain to assure safe access to treatment for patients. One important factor for sharing tissues with similar quality is to harmonise protocols and improve donation and transplantation activities as European financed projects promote (EuroGTPII; GAPP JOINT ACTION; EGALITE).

From the 4088 CV donors, 3115 hearts and 2095 vascular segments were processed. After performing quality control 53% of the AV, 33% of the PV and 27% of the vascular segments were discarded due to morphology, contamination and other reasons (Fig. 4d and f). Similar discard percentages were reported by other TEs. For example between 50 and 60% of heart valves were discarded yearly by EHB (Jashari 2021), while 46% of CV tissues were discarded by the Croatian CVTB (Golemovic et al. 2022). The Canadian vascular graft bank also reported tissue discard rate of 35% after visual inspection (Georges et al. 2021).

Due to the lack of valves for transplant, and always with the aim to give answer to the patients and surgeon needs, the evaluation of older donors raises as a need. As expected, a significant increase in the tissue discard percentage related to tissue quality was observed after the implementation of the age criteria expansion from 66.23 ± 8.81% up to 78.98 ± 3.71% (*p* < 0.05). Regarding heart valves, before implementation of this measure, 41.46 ± 7.17% of AV and 19.61% ± 10.33% of PV valves were discarded. After the change tissue discard increased to 57.29 ± 7.15% and 35.81 ± 6.09% respectively, being the increment statistically significative for both AV (*p* < 0.0005) and PV (*p* < 0.005). Considering donors from 2014 to 2021, and taking into account the age range, it was detected that double tissues were discarded due to tissue quality in older group, 66–70 years old: 49.25% of AV and 12.82% of PV were discarded under 66 years old, while 80.41% of AV and 26.80% of PV were discarded for donors 66–70 years old. Moreover, the percentage of both AV and PV from the same heart discarded due to quality represent an increasing tendency with the age: 3.5% of heart valves under 46 years old were discarded, while this percentace increases to 9.38%, 16.84% and 24.74% in donors between 46 and 55 years old, 56–65 years old and 66–70 years old, respectively. Therefore, the inclusion of older donors has negative impact in the total percentage of tissue discard. In terms of units, after this criteria modification the number of preserved PV increased approximately 10%. These results agreed with previous results published by the German Heart Centre in Berlin, which reported optimal function of the PVs of donors between 65 and 70 years old (Grosse et al. 2008). Another important reason for tissue discard was artery length, which varied considerably during these two decades, with a mean tissue discard rate of 9.41 ± 4.92%. During the last 8 years, PV discard rate due to artery length was 26.12%, while only 0.51% of AVs were discarded for this reason. This fact is related with the previous recover of the lungs for organs transplantation, which requires the pulmonary artery for the solid organ transplant.

Processing practices vary between TE, not only regarding the dissection technique, but also the antibiotics used for tissue decontamination, the cryoprotectants used, or the storage temperature and duration (Heng et al. 2015; Zahra et al. 2019). Decontamination strategy is a controversial issue, reviewed by Germain et al. (Germain et al. 2010,2016) and Jashari et al. (Jashari et al. 2007) concluding that the antibiotic cocktail is more effective when incubated at higher temperatures. In this sense, looking for improving quality in tissues, in 2012, the BTB changed the composition of the antibiotic cocktail and the incubation temperature. This had a positive direct effect on the tissue discard rate due to microbiological contamination, which decreased from 20.34 ± 10.89 to 10.00 ± 3.7% (*p* < 0.05). Also in 2019, the microbiological sampling strategy was updated in order to improve microbiological detection, including liquid sampling during processing, which did not produce any significant changes in tissue discard rates: 10.69 ± 4.96% were discarded due to positive microbiology before this strategy implementation and 9.14 ± 1.33% after it. The EHB also implemented this strategy in 2015 to avoid false negative results (Díaz Rodríguez et al. 2016). In BTB, there were no notification related to infection disease transmission through CV tissue transplantation probably due to the efforts during processing to detect and avoid contamination.

According to EDQM reports, in 2003, 12 Member States reported the distribution of 560 hearts for heart valves and 1136 blood vessesl, while in 2021, 26 Member States reported the distribution of 1322 heart valves and 4356 blood vessels (EDQM Newsletter 2014 and 2021). Interestingly, in Catalonia AV distribution has decreased in the recent years, as reported elsewhere (Jashari et al. 2010), but during 2021 double the grafts were implanted than in 2020, possibly due to the surgeries post-poned due to the COVID-19 outbreak. Comparing aortic and pulmonary valves, as de By et al. commented (de By et al. 2012), the demand for PVs is twice the demand for AV. On the other hand, vascular segment distribution at the BTB can be itimised as follows: 38% femoral arteries, 23% pulmonary/hemipulmonary patches, 20% iliac arteries, 10% aortoiliac bifurcation, and 9% aortas (ascending or descending aorta). This distribution is slightly different from that reported by Jashari, who gave 52% of femoral arteries, 20% aortas and 10% patches (Jashari et al. 2013). The use of cryopreserved arterial homografts for vascular infections has remained constant over the years being a valuable option for the surgical treatment of primary and secondary vascular infections (Mestres et al. 2019). The increase in vascular segments distribution is directly related to the increase in iliac-femoral arteries for by-pass surgery. Even though graft degeneration is described in patients requiring distal revascularisation, patient outcomes and limb survival are good for clinical patient conditions who carry a high morbidity and mortality (González-Gay et al. 2020). The total number of vascular allografts is also influenced by a decrease in the distribution of pulmonary patches used for congenital malformations, probably due the development of novel patch alternatives including biomaterials, decellularisated matrices or 3D bioprinting scaffolds.

Some limitations need to be highlighted. During the last 20 years different IT systems were handled to record the data and different collection criteria were used, depending on the TE and the year. This fact has hampered analysis of the data and comparison of the tend over the evaluated period. Moreover, despite the huge amount of patients treated with CV tissues from the BTB and the efforts made to create a register of valvular and vascular allografts transplants in Catalonia (Guevara-Noriega et al. 2020), the patient follow-up is not shared with the TE, and therefore grafts cannot be properly tracked at the TE. Tissue biovigilance should be encouraged by all TEs and clinicians to enhance the quality of the information available (Zahra et al. 2019). Many publications highlight the successful outcome of homografts. For example, Delmo Walter et al. reported a freedom from re-infection rate after homograft implantation of 91.9 ± 3.6% after 15 years (Delmo Walter et al. 2012). Additionally, Cocomello et al. reported that PVs were associated with a lower transvalvular gradient and a significant lower risk of reoperation or structural valve degeneration during follow-up when compared with bioprostheses (Cocomello et al. 2019). During last 8 years, the distribution of AVs has decreased in Spain by about 50%. This decrease coincides with the implementation of new techniques, for instance the Transcatheter Aortic Valve implantation (TAVI) for the treatment of aortic stenosis (Mack et al. 2015; Thourani et al. 2016) but, certainly, the type of patients could be rather different. During these 20 years, several valvular and vascular substitutes have appeared on the market that are easier to handle and store at room temperature in the OR, with the availability of a broader range of diameters. On the other hand, the preservation, transport and handling of CV homografts still poses a technical and logistical challenge, requiring surgery planning and dry-ice/dry-shipper transport, with limited possibility of devolution to the TE if not used. Moreover, the range of diameters and lengths is limited and does not fully cover the surgical needs. On the other hand, despite there is a renewed interest on operations like the Ross procedure in Europe and the US due to the excellent long term results, those surgical techniques are highly demanding and therefore not for every practitioner. Particularly in Spain, scarce number of surgeons were even trained to do homograft operations and is only now when some groups like the one at HCB are turning to increase the use of homografts, cryo or decellualarized even to be used in the pulmonary position to substitute the pulmonary autograft implanted in the patient’s aorta.

For an already stablished bank, research is crucial. Different ways of decellularization are still under investigation through collaboration with EU projects (ARISE and ESPOIR) or, also very important, new potential processes locally developed. Time of warm and cold iscahemia are also requiring deeper validation to ensure optimal use of the limited amount of tissue. Functional properties of vessels according to the preservation and decontamination is of highest interest in small diameter vessels as the capability of contraction and dilation in such conduits may play, as with the internal mammary artery, a fundamental role in patency at long term. Only investing in research will keep the CV Tissue Banks active.

In conclusion, during the last 20 years, the BTB’s CV bank has implemented specific changes with the aim of increasing donor pool and the processing, storage and distribution of CV tissues, fulfilling patients’ and CV surgeons’ needs and ensuring the safety of the patients. The age expansion had a direct impact on the number of processed donors, but it also had a direct effect on donor and tissue discard. However, after the implementation of this measure the number of preserved tissues increased. On the other hand, the changes in the quality control strategy guarantees better detectability of any possible pathogen, highly improving the safety of the tissue.

## Data Availability

The data sets analyzed during the current study are available from the corresponding author on reasonable request.

## Abbreviations

ART: Arteries
AV: Aortic valve
BST: Banc de Sang i Teixits
BTB: Barcelona Tissue Bank
BV: Biovigilance
CV: Cardiovascular
DC: Donor center
PV: Pulmonary valve
OCATT: Organització catalana de trasplantaments
ONT: Organización nacional de trasplantes
SAE: Serious adverse event
SAR: Serious adverse reaction
TE: Tissue establishment
TSF: Transplant Services Foundation
